# Value and Credibility of Meta-Analysis: Tutorial on Enhancing Methodological Rigor and AI-Powered Efficiency

**DOI:** 10.2196/92132

**Published:** 2026-07-02

**Authors:** Stefano Brini, Tiffany I Leung

**Affiliations:** 1 JMIR Publications Toronto, ON Canada; 2 Department of Internal Medicine (adjunct) Southern Illinois University School of Medicine Springfield, IL United States

**Keywords:** meta-analysis, artificial intelligence, large language models, systematic review, literature review, evidence synthesis, heterogeneity

## Abstract

The value of a meta-analysis is based on its methodological and statistical rigor, yet many published systematic reviews and meta-analyses contain statistical shortcomings that limit their utility for clinical practice and public health. This can make it challenging to aggregate data for treatment choices for patients as well as limit the extent to which policymakers can promote social change and improve public health. This challenge is compounded by the traditionally slow and resource-intensive nature of systematic reviews, which delays the translation of vital evidence. In this tutorial, we address both challenges. We first provide a primer on essential statistical techniques to help authors produce more robust and reliable meta-analyses. We then briefly discuss the growing role of artificial intelligence (AI) in automating tasks in systematic literature reviews and meta-analyses. Ethical use and disclosure of AI in supporting these essential tasks are also important considerations. This guide is intended to help authors enhance the rigor of their work and use new technologies to ensure their findings are both trustworthy and timely.

## Introduction

Meta-analyses are an important part of evidence-based medicine, synthesizing vast bodies of literature and original data to guide clinical practice and shape public health policy. However, their value and credibility are under constant pressure from two major challenges: methodological pitfalls that can generate misleading or statistically unreliable conclusions [1-5] and a laborious, time-consuming process that can delay the translation of critical findings for years [6]. A flawed meta-analysis can misinform clinical guidelines, while a delayed one represents a missed opportunity to improve patient outcomes. This tutorial provides a practical guide for authors to navigate both challenges. First, we will present the essential statistical principles required to ensure the rigor and validity of a meta-analysis, moving beyond software defaults to foster critical thinking (Table 1) [7]. Second, we will explore how artificial intelligence (AI) is changing the efficiency of the systematic review process, creating an opportunity for researchers to produce high-quality evidence faster, transparently, and ethically. We hope to see more high-quality meta-analyses of digital health research published.

**Table 1:** Checklist of the key statistical methods and interpretations for a rigorous meta-analysis.^a^

Area of focus	Key recommendation and rationale	Check [ ]
Protocol registration	Register the protocol prospectively (eg, PROSPERO, OSF). Define the research question and methods beforehand. This reduces outcome reporting bias and ensures any deviations from the original plan are transparently reported.	[ ]
When to combine studies in a meta-analysis	Base the decision to pool data on clinical meaningfulness and research goals rather than statistical homogeneity. Address heterogeneity by using random-effects models and exploring sources of variation through prespecified subgroup analyses or meta-regression.	
Risk of bias (RoB) assessment	Avoid summary quality scores (eg, Jadad scale). Use modern domain-based tools (eg, RoB 2, Risk of Bias In Nonrandomized Studies of Interventions [ROBINS-I]). Prespecify a sensitivity analysis excluding high risk studies to test if the primary results are robust to bias.	[ ]
Model choice	Use a random-effects model. This is typically the correct choice, as it assumes true effects vary across studies, which reflects clinical reality. A fixed-effect model is rarely appropriate and requires strong justification, such as specific, narrow inference questions.	[ ]
Statistical method	Prefer the Hartung-Knapp-Sidik-Jonkman (HKSJ) method. Unlike the default DerSimonian and Laird method, HKSJ produces more reliable confidence intervals and reduces the risk of false-positive findings, especially with few studies or substantial heterogeneity.	[ ]
Heterogeneity assessment	Focus on the prediction interval for interpretation. This metric provides a clinically meaningful range of expected effects in future studies. Report the I² statistic, but do not use it to judge the magnitude of heterogeneity. Moreover, report Q-statistic, tau (τ), and tau-squared (τ²) for a comprehensive description of heterogeneity.	[ ]
Exploring heterogeneity	Base subgroup analyses and meta-regressions on a priori hypothesis. These analyses are observational and prone to spurious findings. Avoid data dredging and ensure a sufficient number of studies for meta-regression (rule of thumb: ≥10 studies per covariate).	[ ]
Small-study effects	Assess for “small-study effects,” not “publication bias.” Use funnel plots and Egger test cautiously, acknowledging they test for a pattern, not its cause. Discuss multiple potential reasons for any observed asymmetry.	[ ]

^a^This checklist includes several elements already present in the PRISMA (Preferred Reporting Items for Systematic Reviews and Meta-Analyses) 2020 expanded checklist [7].

## Part 1: Statistical Rigor

### When to Combine Studies Into a Meta-Analysis

Researchers often hesitate to combine studies due to perceived high heterogeneity [8], sometimes even using the I-squared (*I*²) statistic as a post-hoc filter to discard results. This is a fundamental misunderstanding; requiring perfect similarity across populations, interventions, and methods would render meta-analysis impossible. The random-effects model is designed precisely to synthesize data from this diverse universe of studies to estimate an average effect. Instead of abandoning the analysis when differences arise, authors should view this variation as an opportunity to explore prespecified sources of heterogeneity (eg, disease severity, dose) through subgroup analysis or meta-regression.

Ultimately, the decision to pool data depends on the research goal and clinical meaningfulness [9]. For instance, a review on telehealth for chronic disease management could validly combine studies on diabetes and chronic obstructive pulmonary disease to estimate the broad impact of the intervention. While the conditions differ, the broad question is clinically meaningful. Authors can then preserve the granularity of the data by using subgroup analyses or meta-regression to determine if the effect varies by disease type, rather than assuming the studies are too different to combine at all.

### Protocol Registration and PRISMA Guidelines

True methodological rigor begins before a single search is conducted. We recommend that authors register their systematic literature review (SLR) protocol prospectively in a public registry such as PROSPERO (International Prospective Register of Systematic Reviews) or OSF (Open Science Framework). Registration serves as a permanent record of the intended study methods, outcomes, and analysis plan. This step helps to distinguish confirmatory analyses (planned beforehand) and exploratory analyses (generated after seeing the data). While deviations from the protocol are often necessary, they must be transparently reported and justified to avoid the appearance of “p-hacking” or outcome reporting bias. A meta-analysis without a preregistered protocol carries a higher risk of bias and lower credibility. Authors who registered the protocol before commencing SLR should report any deviations from the published protocol.

Authors will also need to adhere to the PRISMA (Preferred Reporting Items for Systematic reviews and Meta-Analyses) statement [7,10]. There are also several PRISMA extensions that authors are encouraged to use when reporting an SLR. For example, the PRISMA 2020 extension for Abstracts [7] and the PRISMA-Search (PRISMA-S) [11] are particularly useful to ensure sufficient information about the methodology to ensure full transparency and clarity of reporting and to provide sufficient details to readers to be able to replicate the methodology of the review in future. When reporting the search strategy using the PRISMA-S checklist, authors should ensure that if a specific item in the checklist cannot be addressed because it was not part of the research methodology, then this needs to be mentioned in the manuscript rather than marking that particular item as “Not Applicable” in the checklist.

### Assess Input Quality: Risk of Bias

While statistical models address numerical heterogeneity, they cannot correct for the fundamental flaws in the study design. A meta-analysis should also assess the “Risk of Bias” (RoB) of the included studies by using tools such as the Cochrane RoB 2 tool for randomized trials or Risk of Bias In Nonrandomized Studies of Interventions (ROBINS-I) for nonrandomized studies. We recommend prespecifying a sensitivity analysis that restricts the primary meta-analysis to studies with “low” or “moderate” risk of bias. If the pooled effect disappears when high-risk studies are removed, the primary finding may be an artifact of poor study quality rather than a true clinical effect.

### Model Choice: Fixed-Effect vs Random-Effects in Meta-Analysis

The choice between a fixed-effect and a random-effects model in a meta-analysis is a foundational decision that dictates the interpretation of the results [12,13]. A fixed-effect model assumes there is only one true effect size across all studies and that any observed variation is due to sampling error [14,15]. This is analogous to multiple people measuring the height of a single wall; there is one true height, and differences in measurements between raters arise only from measurement errors. Consequently, the inference from a fixed-effect model applies only to the specific set of the included studies. Using this model when true heterogeneity exists can lead to overly precise confidence intervals and inflate the risk of false positives. A fixed-effect model may be appropriate in some instances, like pharmaceutical research, where studies are true replications. For example, if multiple sites in a multi-center clinical trial use an identical protocol, participant source, and outcomes, any variation is likely due to sampling error, not true heterogeneity. In this specific scenario, a fixed-effect analysis is justified [12,13].

A random-effects model, by contrast, assumes the true effect itself varies from one study to the next, conceptualizing the included studies as a sample from a wider universe of potential studies [14]. This is like measuring patient depression levels across different clinics; the intervention's effect will likely differ due to variations in participants’ demographic (eg, age, sex, education level), clinical factors (eg, comorbidities, disease severity), or variations in the intervention such as frequency, intensity, and duration, or different types of digital health applications such as telemedicine or telehealth. The summary estimate therefore represents the average effect in this universe of studies. As most meta-analyses combine studies with such inherent diversity, the random-effects model is generally the more conceptually sound and appropriate choice, allowing for broader generalization of the findings.

It follows therefore, the choice between the fixed-effect and random-effects model is conceptual rather than just statistical [14]. This means that authors should decide which model to use based on whether they think studies share a common effect size or not. In fields such as evidence-based medicine, digital mental health, mobile health, digital health, assistive technologies, infodemiology, neurotechnology, and additional innovation and eHealth topics in scope for JMIR Publications journals, and when pooling studies from the literature, the random-effects model is likely the most appropriate.

### Statistical Method: Prefer Hartung-Knapp-Sidik-Jonkman Over DerSimonian-Laird

Within the random-effects model, the choice of the statistical method is also critical. The default DerSimonian and Laird (DL) method, which was initially developed by the end of the 1980s [16], is ubiquitous in software such as the Cochrane's Review Manager, but when the meta-analysis includes a few studies (eg, <10) or there is substantial heterogeneity, it tends to underestimate the true variance between studies tau-squared (τ²) [17-20]. This underestimation results in confidence intervals that are deceptively narrow, increasing the risk of type I error (ie, concluding an effect exists when it does not) [5].

The Hartung-Knapp-Sidik-Jonkman (HKSJ) method, which was described by the end of the 1990s [21], provides a crucial and well-validated adjustment [3,5,22,23]. It accounts for the uncertainty in the estimation of τ² by using a t-distribution (rather than the normal distribution used by DL), which produces wider, more conservative confidence intervals. This approach has been consistently shown in simulation studies to maintain the correct type I error rate. Given the HKSJ method is better able to control for false positives than the DL method, we strongly recommend that authors move beyond software defaults and adopt the HKSJ method as their primary approach, especially when heterogeneity is present. In several software such as in Comprehensive Meta-Analysis [24] or when coding in R [25], HKSJ can be selected. For extremely small meta-analyses, qualitative synthesis or restricted maximum likelihood analyses may be alternatives [12,20].

### Heterogeneity: The I² and Prediction Intervals

Heterogeneity refers to the genuine differences in effects across studies, and its proper interpretation is paramount. Authors frequently report the *I*² statistic, but often misinterpret it as a measure of the magnitude of variation [26-28]. The *I*² only describes the proportion of the total variation due to true between-study differences rather than sampling error. An *I*² of 90% simply indicates that most of the observed variability is real, but it does not tell us if the effects are clinically different, they could all be large and beneficial, or they could range from beneficial to harmful [14,28,29]. Authors often interpret an *I*² of 90% for example, as indicating high heterogeneity. But an *I*² = 90% simply indicates that 90% of the observed variation of effect sizes is due to true differences among studies rather than sampling error; it does not provide information on which population or setting the treatment is beneficial, null, or harmful [26-29]. As such, authors should avoid qualifying indices of heterogeneity as low, medium, or high but simply reporting as a descriptive index without making further inferences.

A more clinically informative metric is the prediction interval [15,30]. It estimates the range where the true effect size in a future, similar study is expected to lie, providing a tangible sense of the expected variability in practice [29]. Its power is in its interpretation: a meta-analysis of a new drug might yield a pooled odds ratio with a 95% CI of 1.2-1.8, suggesting an average benefit. However, the 95% prediction interval could be 0.7-3.1. This reveals a profoundly different clinical picture: while beneficial on average, in some settings the drug may be ineffective or even harmful (odds ratio < 1.0). This is vital for clinical decision-making and for understanding the generalizability of the results [15,26,27,29].

As such, prediction intervals should be routinely reported in meta-analyses that include a sufficient number of studies—generally 10 studies or more [28-30]. When a meta-analysis includes fewer than 10 studies, bootstrapping can be used to calculate reliable prediction intervals [31]. The R-package *pimeta* can be used to produce prediction intervals using bootstrapping [32]. Moreover, to provide a comprehensive description of heterogeneity, other indices of heterogeneity, including the Q-statistic, tau (τ), and τ² should also be reported [14]. The Q-statistic is an index of homogeneity under the null hypothesis that studies in the meta-analysis share a common effect size, while τ² is an index of between-study variance and τ is the square root of τ² (ie, the standard deviation of the true effect size).

### Exploring Heterogeneity: Subgroup Analysis and Meta-Regression

When substantial heterogeneity exists in a meta-analysis, authors should investigate its sources by using prespecified analyses to avoid spurious findings. Subgroup analysis compares pooled effect estimates between different groups of studies such as those with high vs low risk of bias or different patient populations [30]. For example, a meta-analysis might compare the pooled effect from 10 randomized controlled trials (RCTs) in patients with a mild form of a disorder against the pooled effect from 10 RCTs in patients with a severe form. The goal is to test if the treatment effect is significantly different between these two subgroups.

Critically, even when analyzing RCTs, the subgroup comparison itself is observational. The randomization that occurred *within* each trial does not apply to the *comparison between* the subgroups of studies. Therefore, any findings are associative, not causal. Authors must use a formal statistical test for subgroup differences (eg, the Q-statistic) but should be aware that these tests are often underpowered, meaning a nonsignificant result does not rule out a true difference between the groups. Authors should report the Q-statistic, which is an omnibus test (it does not indicate which of the subgroups differ against one another, ie, it tests the null hypothesis that all subgroups are equal), its degree of freedom, and *P* value.

Meta-regression explores the relationship between a continuous study-level characteristic (a covariate, such as average patient age, medication dose, or duration of follow-up) and the intervention effect [33]. Although there is no specific number of studies that should be included for each study-level covariate in meta-regression, the Cochrane Handbook suggests having at least 10 studies for each covariate included in the model to minimize the risk of false-positive findings [34]. This is because increasing the number of covariates, particularly in meta-analyses with few studies, is associated with increased risk of false-positives among selected covariates [35]. Both methods are observational by nature; they can identify associations, but they cannot establish causation. Therefore, these investigations should be based on a small number of strong, a priori hypotheses grounded in clinical plausibility, not on post-hoc data dredging.

### Small-Study Effects vs Publication Bias

Authors often incorrectly state that funnel plots and Egger test assess publication bias. These tools do not, and cannot, directly test for the suppression of null-finding studies [13,36,37]. Instead, they test for small-study effects: a statistical pattern where smaller studies show systematically larger effects than larger studies [37,38]. Visually, this appears as an asymmetrical funnel plot, with a gap in one of the bottom corners [37,39]. While publication bias may be one source for this pattern, it is not the only cause. Small-study effects can also arise from other factors [38,40]. For example, smaller studies may have less methodological rigor [41], or they might enroll higher-risk patients who genuinely exhibit larger treatment effects. It is also possible that interventions are implemented with greater fidelity in smaller, more controlled trials. Therefore, authors should report evidence of funnel plot asymmetry as a “small-study effect” and transparently discuss the multiple possible explanations. Even if these tests are negative, authors should still acknowledge the pervasive risk of publication bias within their field as a general limitation of the available evidence.

## Part 2: Accelerating the Systematic Review Process With AI

### Integrating AI in Evidence Synthesis and Meta-Analysis

**Figure 1:**
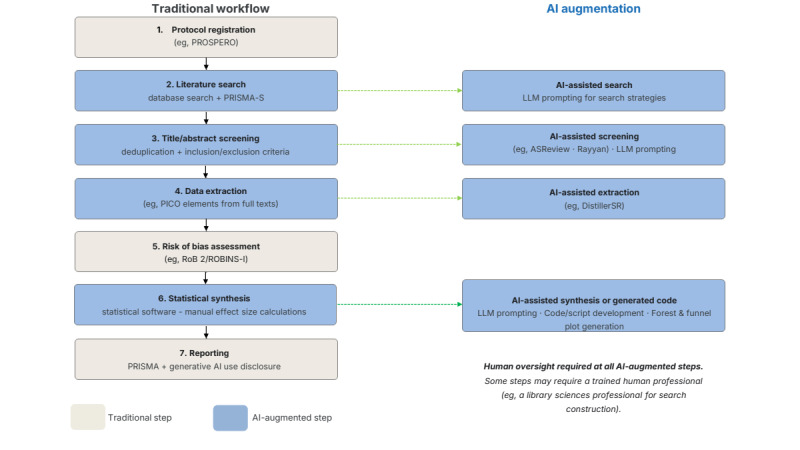
Schematic summarizing the traditional meta-analysis workflow with possible AI-augmented steps. AI: artificial intelligence; LLM: large language model; PICO: Population, Intervention, Comparator, and Outcome; PRISMA-S: Preferred Reporting Items for Systematic Reviews and Meta-Analyses Search; RoB: risk of bias; ROBINS-I: Risk of Bias In Nonrandomized Studies of Interventions.

While applying the statistical techniques above is essential for validity, the sheer scale of the SLR process presents another major hurdle. The end-to-end traditional SLR process is a notoriously laborious and resource-intensive endeavor, often taking years to complete [6]. AI could be a powerful aid for increasing the efficiency of evidence retrieval and synthesis [42]. Moreover, AI can assist researchers in conducting meta-analyses using the latest statistical techniques. For instance, it can generate the necessary R scripts for sophisticated modeling, thereby strengthening the study's overall statistical rigor. Researchers are increasingly using various forms of AI to accelerate the delivery of evidence to clinicians and policymakers [43-46], and academic and industry services are increasingly incorporating more sophisticated technologies into their products (Figure 1). Chief among these is generative AI (GAI), which is a type of AI capable of extracting data and generating novel content such as text, images, and summaries from previously learned patterns.

While early AI models sometimes struggled with specific tasks like extracting numerical data, the technology is rapidly advancing. Tools using machine learning are routinely used for critical, time-consuming steps like literature deduplication and screening [43]. For example, AI successfully screened over 60,000 articles in ecology [47]—a task that would be significantly time-consuming for human reviewers. As of June 2024, 172 studies had been identified as automating at least one literature review task [48], yet only 2 articles had focused on the entire cycle of review supported by large language models (LLMs) [49,50]. Comparison of human-conducted vs LLM-prompted literature review on one medical subject suggested that the main benefits of GPT-4 prompted (ie, guiding the LLM through an iterative process that recurrently refines initially broad instructions into a more precise final prompt based on continuous evaluation) literature search, screening, and data synthesis, including breadth of content, faster processing, and reasonable accuracy; however, drawbacks included lack of transparency, lower consistency, and, importantly, limited contextual understanding [51]. One SLR suggested that GAI to help with creating a search strategy can result in a higher number of search results, yet still miss retrieving relevant articles [52]. Consequently, if AI is used to generate search strings, we strongly recommend that the output still be validated by human experts against PRESS (Peer Review of Electronic Search Strategies) guidelines to ensure no critical concepts are missed due to AI-hallucinated syntax [53]. As a final note, consulting a professionally trained librarian or information retrieval specialist to build a robust search strategy still remains a gold standard: GAI may augment but so far cannot substitute for this important library service [54].

Another study suggested that LLM-assisted literature screening (ChatGPT-4o and Claude-3.5 Sonnet) outperformed machine learning–based tools (ASReview and Abstrackr) for the same SLR task [55]. In particular, again data extraction including “assessing nuances that necessitates drawing inferences” was a difficult task for GPT-4 [56], although GPT-4 making reasoning errors and avoiding applications in “high-stakes contexts” is explicitly acknowledged by OpenAI [57]. At the time of this article's revision, systematic review platforms are applying AI assistance in tasks such as screening (ASReview [58] and Rayyan [59]), deduplication (Rayyan and Covidence [60]), and data extraction (DistillerSR [61]). There are many more tools with variations on their applications of AI assistance even for the same tasks. Providing a comprehensive synthesis of the sensitivity and specificity for every possible AI-assisted review tool and their technologies is beyond the scope of this tutorial.

In current times, specific data extraction subtasks may be performed by AI more accurately and faster than humans [43,62]. One study using GPT-4o, an LLM, found that it could extract Population, Intervention, Comparator, and Outcome (PICO) data from over 680,000 abstracts with 98% accuracy [63]. LLMs have demonstrated high accuracy (up to 99%) in extracting predefined data points from primary studies and have even generated the necessary R scripts for network meta-analysis [64]. However, it is possible that key limitations of GAI specifically for data extraction will be limited by the complexity of the datasets in the original research included and tool-specific limitations [65]. Furthermore, researchers are exploring AI's potential in additional SLR analytic tasks, such as conducting risk of bias assessments and creating plain language summaries of review findings. In these advanced applications, AI may serve as a powerful assistant, although for now, human oversight remains crucial to verify accuracy and context, especially for the specific task delegated to AI during the study methodology.

AI assistance in evidence synthesis raises important questions regarding transparency, reproducibility, and bias propagation. The speed of AI tool development may amplify biases that already exist, even in human-conducted evidence synthesis. To prevent this, the application of necessary checks and balances could help to ensure the transparency [66], integrity, and accuracy of researchers’ findings.

### Transparency and Disclosure

Authors who conduct SLR and meta-analyses inevitably use software that assists with at least organizing their literature. As AI technologies evolve and become increasingly integrated into products to assist this study methodology, providing detailed methodological descriptions ensures adherence to scientific norms of reproducibility, transparency, and integrity. Practically, this means that authors are expected to include in the Methods section a sufficiently detailed explanation of tasks supported by software or AI tools and citation of all tools used to assist the SLR and meta-analysis reported.

Authors are strongly encouraged to scrutinize the validity of various tools before conducting their study. Commercial review-assistance products and commercial LLMs vary in their transparency and disclosure of how such tools work. For example, commercial software may claim their usefulness in a review methodology, yet they may not explicitly state that they use LLMs as a core technology. Some products also claim to offer “AI-assisted” or “AI-powered” solutions for specific literature review tasks, yet may not provide adequate algorithmic transparency or evidence to support such a claim, and may not hold up to rigorous evaluation against published human-performed SLR [65]. Given the limitations of LLMs in performing certain review and analysis tasks, authors’ transparency in their use of tools is essential in strengthening the credibility and integrity of their scientific work. To ensure reproducibility, authors should specify the exact model and version (eg, GPT-4o, Gemini 1.5 Pro, etc) and preferably include the date of access, as capabilities evolve rapidly [67]. Reporting the system prompts or interaction logic is also crucial. Authors may further enhance rigor by using multi-turn interaction strategies by asking the model to review, critique, and refine prior output. This reduces hallucinations (factually erroneous but plausible outputs) and improves overall response quality. Although some scholars argue the term “confabulation” [68] or “falsification” [69] better reflects the AI's underlying mechanisms, preventing these inaccuracies is critical. In evidence synthesis, misleading conclusions can directly translate into harmful real-world outcomes for patients and the public.

At the time of this tutorial, we recommend against using GAI as the sole conductor of statistical analyses (eg, asking a chatbot to calculate an odds ratio) due to the risk of mathematical hallucinations [70]. However, authors should distinguish this from using GAI for code generation. Asking an LLM to write syntax (eg, R or Python scripts) for an analysis is a potentially valuable efficiency aid because the output is verifiable: the code can be reviewed for logic and executed in standard statistical software to produce reliable, reproducible results. If applied for code generation, this must be human-verified (author is accountable), the method described, and the usage disclosed (work is transparently reported) [67]. Additionally, if authors wish to use commercially available LLMs to help them check their adherence to reporting guidelines, such as PRISMA [7] and PRIOR [71], they should be cautious due to the low reported accuracy of some LLMs in assuring such reporting guideline adherence [72].

Author requirements on disclosure of GAI use are journal-dependent, with many journals requiring disclosure. If authors describe GAI use precisely and thoroughly in the Methods section of the manuscript, then additional publishable disclosure statements may not be required. Authors must check journal policies early on in their research process to ensure their study design and steps on preparing a manuscript are compliant; recordkeeping akin to keeping a detailed lab notebook, for example, retaining prompts and responses, is strongly recommended, as this supports potential reproducibility and transparency. Ultimately, the key principles of accountability, transparency, and confidentiality must be adhered to throughout manuscript materials and GAI use disclosure statements [67].

### Human Expertise and AI

The credibility of a meta-analysis hinges on rigorous statistical methods, which the researcher can learn and use responsibly. This requires moving beyond software defaults to make deliberate choices: applying robust analytical methods like the HKSJ correction, selecting appropriate meta-analytic models, and interpreting clinical relevance through tools like prediction intervals. Executing this level of detailed analysis, however, is often hampered by the time-consuming nature of the SLR process. GAI may offer a transformative solution; however, appropriate understanding of their limitations and human oversight are required to ensure the integrity of conducted research.

## Appendix

Multimedia Appendix 1 Screenshots of the prompts and responses for Claude Sonnet 4.

## Abbreviations

AI: artificial intelligence
DL: DerSimonian and Laird
GAI: generative artificial intelligence
HKSJ: Hartung-Knapp-Sidik-Jonkman
LLM: large language model
PICO: Population, Intervention, Comparator, and Outcome
PRESS: Peer Review of Electronic Search Strategies
PRISMA: Preferred Reporting Items for Systematic Reviews and Meta-Analyses
RCT: randomized controlled trial
RoB: risk of bias
ROBINS-I: Risk of Bias In Nonrandomized Studies of Interventions
SLR: systematic literature review

## Data Availability

Data sharing is not applicable to this article as no data sets were generated or analyzed during this study.
