# Supplementary figures and images for: Value and Credibility of Meta-Analysis: Tutorial on Enhancing Methodological Rigor and AI-Powered Efficiency
Source: J Med Internet Res. 2026 Jul 2;28:e92132. doi: 10.2196/92132 (PMC13376850; doi:10.2196/92132)

Screenshots of prompts and responses for Claude Sonnet 4.6

27-Apr-2026


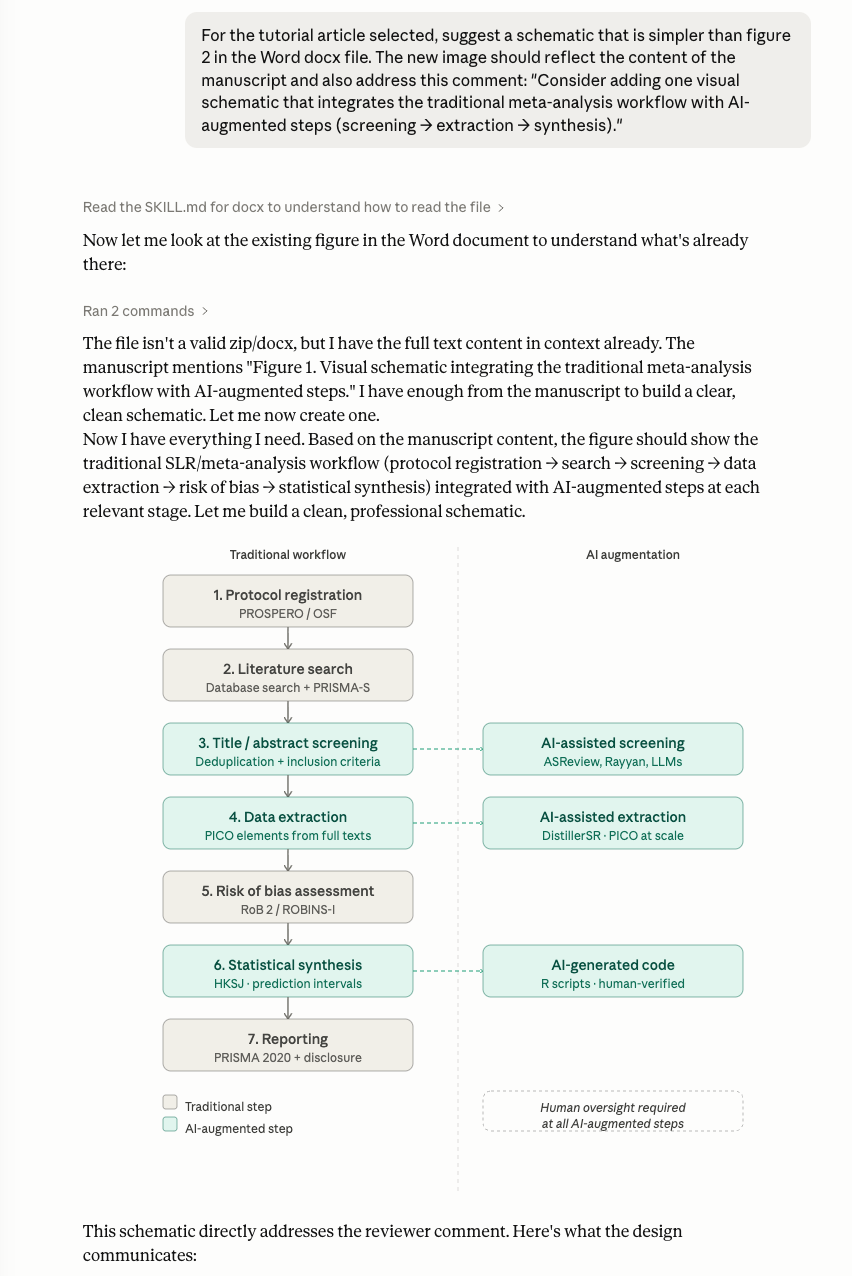


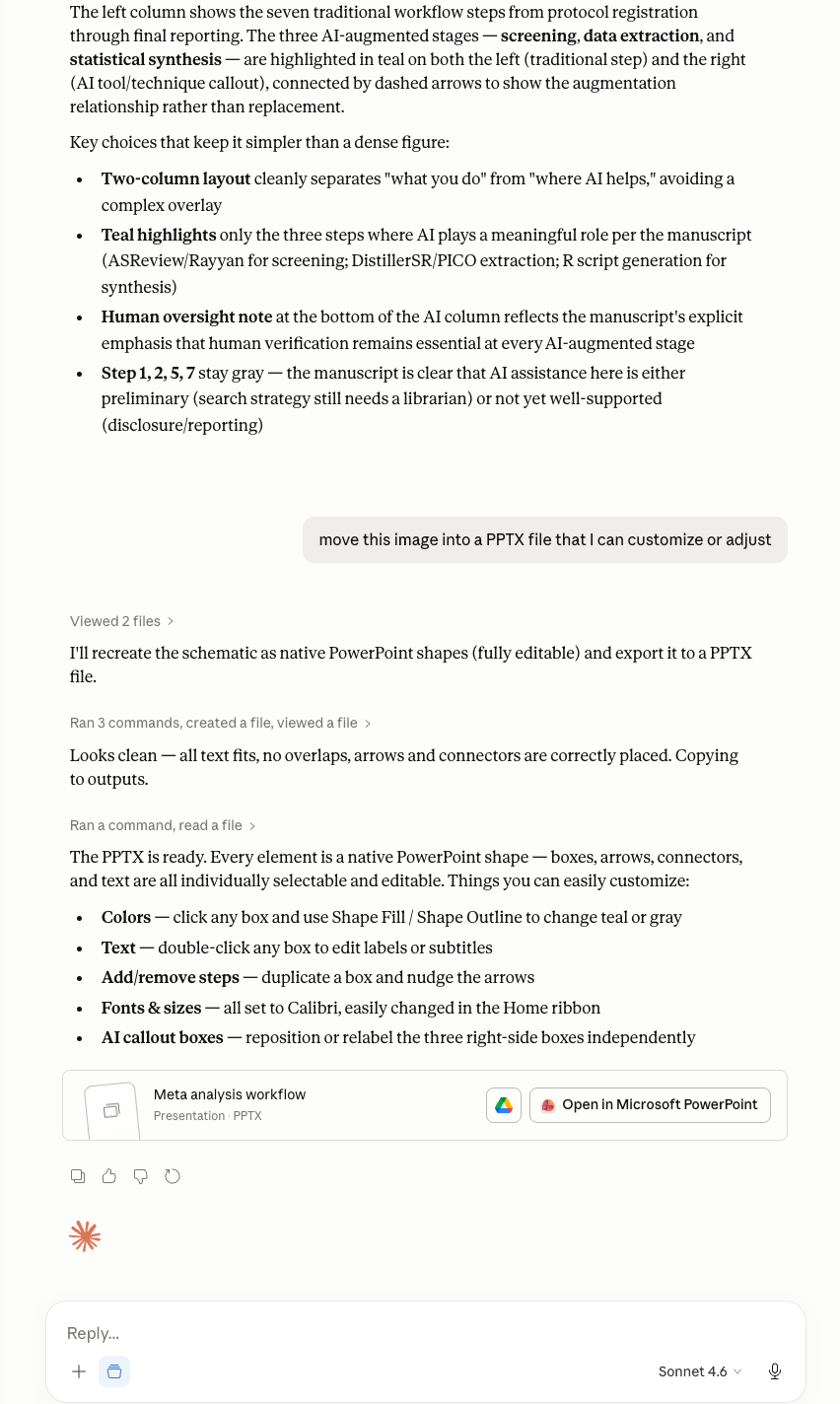

Supplement: Multimedia Appendix 1 [file jmir_v28i1e92132_app1.docx]
